# Ebola virus disease: assessment of knowledge, attitude and practice of nursing students of a Nigerian University

**DOI:** 10.4314/ahs.v18i1.9

**Published:** 2018-03

**Authors:** Aniekan J Etokidem, Boniface U Ago, Mary Mgbekem, Affiong Etim, Eno Usoroh, Anastasia Isika

**Affiliations:** 1 Department of Community Medicine, University of Calabar, Calabar, Nigeria; 2 Department of Community Medicine, University of Calabar Teaching Hospital, Calabar, Nigeria; 3 Department of Obstetrics and Gynaecology, University of Calabar, Calabar, Nigeria; 4 Department of Obstetrics and Gynaecology, University of Calabar Teaching Hospital, Calabar, Nigeria; 5 Department of Nursing Science, University of Calabar, Calabar, Nigeria; 6 Department of Physiology, University of Calabar, Calabar, Nigeria; 7 KNCV-Nigeria/ChallengeTB, Akwa Ibom State, Nigeria

**Keywords:** Ebola, infection, virus, disease, haemorrhagic, fever, Nigeria

## Abstract

**Background:**

Ebola virus disease has a high case fatality rate. Health care providers have a key role to play in its management and prevention.

**Objectives:**

The aim of this study was to determine the knowledge, attitude and practice regarding Ebola virus disease (EVD) among students of Nursing in the University of Calabar, Calabar, Nigeria.

**Methods:**

This was a descriptive cross-sectional study. A semi-structured,self-administered questionnaire was administered to 178 nursing students. The respondents were selected using systematic random sampling.

**Results:**

Lecturers were the commonest source of information regarding EVD as reported by 84.5% of the respondents followed by television (76.3%). Nearly 27% of respondents indicated that the fear of EVD had dampened their interest and enthusiasm in the nursing profession. Nearly 12 % indicated that they would not take part as theatre nurses in the surgical operation of a patient who had been certified cured of EVD. A similar proportion indicated their unwillingness to attend to a woman in labour even if she had been certified cured of EVD.

**Conclusion:**

Majority of the students were knowledgeable about EVD. Although majority of them had favorable attitude towards EVD patients, there was evidence of stigmatizing and discriminatory attitude that needs to be addressed.

## Introduction

Ebola virus disease (EVD), which was formerly known as Ebola haemorrhagic fever (EHF), first appeared in 1976 in 2 simultaneous outbreaks; one in present day Nzara in South Sudan and the other in Yambuku, Democratic Republic of Congo. The latter occurred in a village near the Ebola River, after which the disease was named.^1^,^2^ Between 1976 and 2013, up to 20 outbreaks had been reported, resulting in about 2500 cases in 6 countries namely, the Democratic Republic of Congo, Sudan, Gabon, Côte d'Ivoire, Uganda and the Republic of the Congo.^3^ EVD is caused by a virus of the family *Filoviridae*, genus *Ebola virus*. The genus *Ebola virus* is divided into five different species namely *Zaire, Sudan, TaiForest, Bundibugyo*, and *Reston* viruses. The virus is highly virulent in humans although each genus has varying degree of virulence. Fruit bats and non-human primates constitute the natural reservoir of the virus. Transmission of the virus is through contact with blood, body secretions and fluids such as milk, semen as well as tissues of infected persons either while they are alive or immediately after their death. The disease has an incubation period of 2 to 21 days.^4^

The case fatality rate could be as high as 90%, especially for the *Zaire Ebola virus* which is the most fatal.^5^,^6^ The symptoms of the disease include fever, asthenia, diarrhoea, headache, vomiting, abdominal pain, sore throat, joint aches, conjunctivitis muscle aches and chest pain.^5^,^6^,^7^,^8^

West Africa experienced an outbreak of EVD in January 2014 starting in Guinea and spreading to Sierra Leone and Liberia. Within months, it had spread to Mali, Senegal and Nigeria. By the end of December, 2014, there were 20,200 confirmed cases of EVD reported with Guinea reporting 2707 cases and 1708 deaths, giving a case fatality rate of 63.1%. Liberia had reported 8018 cases with 3423 deaths, giving a case-fatality rate of 42.7%. Sierra Leone had reported 9446 cases with 2758 deaths, giving a case-fatality rate of 29.2%. Three West African countries that also reported confirmed cases include Nigeria (20 cases with 8 deaths, case fatality rate 40%), Mali (8 cases with 6 deaths, case-fatality rate 75%) and Senegal (1 case, no death, case fatality zero).^9^

The first case of EVD was introduced into Nigeria on 20^th^ July 2014 when the diagnosis was made in a Lagos hospital in a Liberian diplomat whose journey to attend a conference in Calabar, Cross River State of Nigeria, was aborted due to the illness and his subsequent death. Subsequently, a chain of transmission was set off that infected a total of 20 people, 8 of whom died of the infection.^9^,^10^

Health care providers, by virtue of the likely exposure to the blood and body fluids of their patients in the course of providing care, are at risk of contracting the disease from their clients, especially if they do not observe standard precautions and use personal protective equipment.

Study among students in different parts of the world have shown interesting results. A study in Malaysia found that the current level of knowledge about EVD among students and staff studied was low (median knowledge score <50%)^11^. The study also found that science-based students had more knowledge than arts and social science-based students (median score = 46.2%, *P* < 0.0001). A similar study among University students in India found that 66.6% of the students had inadequate knowledge, 26.6% had moderate knowledge and 6.6% had adequate knowledge. Regarding the transmission of EVD, a study among college students in the USA found that 88% percent of them knew that the Ebola virus can be transmitted through “body fluids of an infected person,” but 31% believed the virus can be transmitted through mosquitoes. 12 A study in Lagos, Nigeria, found that 67% of the sampled health care workers had good knowledge of the Ebola virus disease, about 33% had fair (average) knowledge while 6 percent had poor knowledge.^13^

## Aim of the study

The aim of this study was to determine the knowledge, attitude and practice regarding Ebola virus disease among students of Nursing in the University of Calabar, Calabar, Nigeria.

## Objectives of the study

The specific objectives of the study were:

1. To determine the knowledge of nursing students regarding EVD.
2. To determine the attitude of nursing students regarding EVD.
3. To determine the practice of nursing students regarding EVD.

## Methods

### Study setting

The University of Calabar is a second generation Nigerian University founded by the Federal Government of Nigeria in 1975. It is located in Calabar, the capital of Cross River State of Nigeria. The University currently has 15 faculties and three institutes. The Department of Nursing is in the Faculty of Allied Medical Sciences.

**Study design:** This was a descriptive, cross-sectional study.

Sample size determination: The sample size was calculated using the Leslie Kish formula:

n=Z^2^pq/d^2^

Where

n= minimum sample size

Z=standard normal deviate which equals 1.96 at the 95% level of significance.

P= proportion of the desired attribute, which =0.88 representing the proportion of students who were found to have good knowledge of EVD in an earlier study^12^

q=1-p

d=degree of accuracy which is 0.05.

Thus, the sample size was calculated to be 162. Making provision for 10% non-response rate, the sample size became (162+16) =178.

**Sampling procedure:** The Department of Nursing was purposively selected to participate in this study. Systematic random sampling was then used to select individual students to participants in the study.

## Results

### Socio-demographic variables

One hundred and sixty-seven (94.4%) of the participants were females while 10 (5.6%) were males. One hundred and seventy-two (97.2%) respondents were Christians while 5 (2.8%) belonged to other religions. Almost 50% of respondents were in their third year of study while 13% were in their final (5th) year. (Table 1

**Table 1:** socio-demographic characteristics

VARIABLE	FREQUENCY	PERCENT

**Sex**		
Male	10	5.6
Female	167	94.4
**Age group**		
<20	85	48.0
20–24	43	24.3
25–29	23	13.0
30–34	23	13.0
>34	3	1.7
**TRIBE**		
Efik/Ejagham/Bewkwarra	36	20.3
Ibibio/Annang/Oron	36	20.3
Igbo	53	30.0
Others	52	29.4
**RELIGION**		
Christianity	172	97.2
Islam	5	2.8
MARITAL STATUS		
Single	160	90.4
Married	17	9.6
**YEAR OF STUDY**		
3	88	49.7
4	66	37.3
5	23	13.0

### Source of information regarding EVD

Lecturers were the commonest source of information regarding EVD as reported by 84.5% of the respondents followed by television (76.3%). Other sources of information regarding EVD were fellow students (37.3%), family members (9.6%), radio (41.8%), Newspapers (24.3%), internet (36.2%), medical journals (11.9%) and medical textbooks (11.3%). (Table 2)

**Table 2:** Source of information regarding Ebola virus disease

Source	Frequency*	Percentage*

Fellow student	66	37.3%
Family member	17	9.6%
Radio	74	41.8%
Television	135	76.3%
Newspaper	43	24.3%
Internet	64	36.2%
Medical journal	21	11.9%
Medical textbook	20	11.3%
Lecturer	150	84.5%

* Multiple responses allowed.

### Knowledge regarding EVD

Over 66% of respondents indicated that the disease was named after a river in the Democratic Republic of Congo. Nearly 68% of respondents identified the monkey as the natural host of *Ebola virus* while chimpanzee was indicated by 27.7%, gorillas (35.6%), fruit bats (39.5%) and swine (11.9%). Regarding the mode of transmission of *Ebola virus*, 71.8% of respondents indicated direct contact with blood and other body fluids and secretions of infected persons while others indicated sexual intercourse (29.4%), preparing and eating infected bush-meat (58.8%), studying in the same room with someone who has not manifested symptoms of EVD (7.9%) and shaking hands with someone who has not manifested symptoms of EVD (7.9%). (Table 3)

**Table 3:** Ebola related general knowledge

KNOWLEDGE VARIABLE	FREQUENCY	PERCENT

ORIGIN OF THE NAME EBOLA		
Named after Dr. Ebola, the first physician who diagnosed it.	28	15.8%
It was named after the village in Liberia	33	18.6%
It was named after a river in the Democratic Republic of Congo	116	65.6%

KNOWLEDGE OF NATURAL HOST OF EBOLA VIRUS		
Monkey	120	67.8%
Chimpanzee	49	27.7%
Gorillas	63	35.6%
Fruit bats	70	39.5%
Forest antelope	75	42.4%
Porcupine	22	12.4%
Swine	21	11.9%

KNOWLEDGE OF MODE OF TRANSMISSION OF EBOLA VIRUS		
Through fomites	14	7.9%
Contact with blood and body fluids/ secretions of infected persons	127	71.8%
Sexual intercourse	52	29.4%
Transfusion of infected blood	64	36.2%
Preparation and eating of infected bush-meat	104	58.8%
Shaking hands with someone who has not manifested symptoms	14	7.9%
Studying in the same classroom with someone who has not manifested symptoms of EVD.		
INCUBATION PERIOD OF EBOLA VIRUS DISEASE	14	7.9%
1 to 7 days	31	17.5%
2 to 21 days	124	70.1%

WHICH COUNTRY REPORTED FIRST CASE IN THE WORLD?		
Liberia	85	48.0%
Democratic Republic of Congo	71	40.1%

WHEN WAS THE FIRST CASE REPORTED IN NIGERIA?		
January, 1999	11	6.2%
April, 2004	21	11.9%
July 2014	109	61.6%

KNOWLEDGE OF SYMPTOMS OF EBOLA VIRUS DISEASE		
Fever	120	67.8%
Fatigue	67	37.9%
Joint and Muscle pain	117	66.1%
Headache	127	71.8%
Sore throat	67	37.9%
Nausea and vomiting	89	50.3%
Internal and external bleeding	163	92.1%
Redness of the eyes	46	26.0%
Rashes	21	11.9%
Polyuria	21	11.9%

Regarding the incubation period of EVD, 70.1% of respondents indicated that it is between 2 to 21 days while 17.5% indicated that it is 1 to 7 days. Forty-eight percent of respondents indicated that the first case of EVD in the world was reported in Liberia while 40.1% indicated that it was reported in the Democratic Republic of Congo. Nearly 62% of respondents indicated that the first case of EVD in Nigeria was reported in July 2014 while about 12 % indicated that it was in April, 2004. Majority of respondents, 92.1%, indicated that internal and external bleeding are symptoms of EVD. Other symptoms indicated are fever (67.8%), joint and muscle pains (66.1%), headache (71.8%) and nausea and vomiting (50.3%) amongst others. (Table 3)

Regarding treatment of EVD, 71.8% of respondents indicated that there was no known cure yet, 15.8% reported that it could be treated with antibiotics like ampicillin while 12.4% indicated that only spiritual cure was recommended. Concerning prevention of EVD, 80.2% of respondents indicated that patients should be treated in isolation centers, a similar proportion indicated regular hand washing and avoiding contact with blood and body fluids of EVD patients. Addressing myths and misconceptions was the preventive measure indicated by 15.8% of respondents while 12.4% indicated taking chemoprophylaxis each time one touched a suspicious patient. (Table 3)

### Attitude regarding EVD

One hundred and fifty-six (88.1%) respondents indicated that the outbreak of EVD had put fear in them, 26.5% indicated that the fear of EVD had dampened their interest and enthusiasm in the nursing profession while 30.5% indicated that they no longer felt comfortable traveling in public transport during the outbreak. Similarly, 12.4% indicated that they were no longer comfortable siting with fellow students in the classroom and 26.6% indicated that they were no longer comfortable going to the hospital ward. Over 72% of respondents indicated that they would encourage a roommate suspected of having EVD to go to the hospital, 24.3% indicated that they would pack out of the room and never return even after such roommate had been declared cured of EVD while 7.9% indicated that they would pack out and return to the room after the roommate had been declared cured of EVD. All (100%) respondents indicated that they would continue to befriend someone who had been certified cured of EVD while 40.1% indicated that they would not eat food from a school food vendor who had EVD but had been certified cured. Similarly, 12.4% of respondents indicated that they would not take part as theatre nurse in the surgical operation of a patient who had been certified cured of EVD. (Table 4)

**Table 4:** Attitude toward people with EVD

WHAT WOULD YOU DO IF YOUR ROOM MATE IS SUSPECTED TO HAVE EBOLA VIRUS DISEASE?	Frequency		Percent	

I would encourage her to go to the hospital	128		72.3%	
I would pack out of the room and never return even after she is declared cured.	43		24.3%	
I would pack out of the room and return after she has been declared cured	14		7.9%	
I would call the ebola hotline	90		50.8%	
I would first buy medicine from the chemist for her and encourage her to go to the hospital if the problem persists.	22		12.4%	

DICHOTOMOUS ATTITUDE RELATED VARIABLES	YES		NO	

If your friend who had EVD has been certified cured of the disease, would you be willing to continue to be his/ her friend?	177	100%	0	0%
If a food vendor in the school cafeteria is known to have had EVD but is now certified cured, would you still eat the food she prepares?	92	52.0%	71	40.1%
If you know that your patient had EVD but has been certified cured, would you take part in his or her surgical operation as the theatre nurse?	155	87.6%	22	12.4%
If you know that your patient who has come in labour had EVD but has been certified cured, would you deliver her of her baby?	135	76.3%	21	11.9%

### Practice regarding EVD

All (100%) respondents indicated that the outbreak of EVD caused them to increase the frequency with which they washed their hands. All respondents also indicated that the outbreak caused them to use personal protective equipment more often than before. Similarly, all respondents indicated that the outbreak caused them to carry their personal hand sanitizers. Only 22 (12.4%) of respondents indicated that they would encourage their friends and family members to be volunteers in EVD vaccine trial just as 103 (58.2%) respondents indicated their willingness to be volunteers in such a trial. Only 66 (37.3%) of respondents had attended a workshop or training in EVD prevention. (Table 5)

**Table 5:** practice regarding EVD prevention

PRACTICE VARIABLE	RESPONSE			

	YES		NO
Did the outbreak of EVD cause you to increase the frequency with which you washed your hands?	177	100%	0	0%
Did the outbreak of EVD cause you to use Personal Protective Equipment (e.g. hand gloves) more often than you used to?	177	100%	0	0%
Did the outbreak of EVD cause you to use hand sanitizer more frequently?	177	100%	0	0%
Did you carry with you your own hand sanitizer during the outbreak in Nigeria?	142	80.2%	21	11.9%
Did you write down or store in your phone any phone number of relevant agencies or organizations or persons to contact in case you suspected that you or someone you know had EVD?	119	67.2%	58	32.8%
Would you be willing to be a volunteer in EVD vaccine trial?	103	58.2%	74	41.8%
Would you also encourage your friends and family members to be volunteers in EVD vaccine trial?	22	12.4%	124	70.1%
In the event of an EVD outbreak in Nigeria, would you be willing to work in the EVD treatment/isolation center?	96	54.2%	81	45.8%
Have you attended any workshop/ training in EVD prevention?	66	37.3%	111	62.7%
Have you attended any workshop/ training in EVD case management?	57	32.2%	120	67.8%
Do you think you still need further information/training regarding EVD	163	92.1%	14	7.9%

### Test of association

Association between students' year of study and some knowledge variables.

As shown in Table 6, although more students in year three, 64 (36.16%) were likely to know the origin of the name Ebola than students in year four, 40(22.6%) and year five, 12(6.7%), the difference was not statistically significant (X^2^= 4.543, df=2, p=0.1031). More students in year three, 66(44.59%) were likely to know when the first case of Ebola Virus disease was diagnosed in Nigeria than students in year four, 31(20.95%) and those in year 5 (12 (8.11%). The difference was statistically significant (X^2^=42.623, df=2, p=0.0000)

**Table 6:** Association between students' year of study and some knowledge variables

Year of study	Origin of the name Ebola		Total	Chi Square	Df	P-value
	Correct	Wrong				
**3**	64(36.16%)	24(13.56%)	88(49.72%)	4.543	2	0.1031
**4**	40(22.60%)	26(14.69%)	66(37.29%)			
**5**	12(6.78%)	11(6.21%)	23(12.99%)			
**Total**	116 (65.54%)	61(34.46%)	177(100.00%)			
**Year of study**	Where first case of Ebola in the world was reported					
**3**	28(15.82%)	60(33.90%)	88(49.72%)	5.205	2	0.0740
**4**	31(17.51%)	35(19.77%)	66(37.29%)			
**5**	12(6.78%)	11(6.21%)	23(12.99%)			
**Total**	71(40.11%)	106(59.89%)	177(100.00%)			
**Year of study**	When first Nigerian case was diagnosed			42.623	2	0.0000
**3**	66(44.59%)	0(0.00%)	66(44.59%)			
**4**	31(20.95%)	28(18.92%)	59(39.87%)			
**5**	12(8.11%)	11(7.43%)	23(15.54%)			
**Total**	109(73.65%)	39(26.35%)	148(100.00%)			
Year of study	Incubation period of EVD			7.178	2	0.0276
**3**	49(31.61%)	17(10.97%)	66(42.58%)			
**4**	52(33.55%)	14(9.03%)	66(42.58%)			
**5**	23(14.84%)	0(0.00%)	23(14.84%)			
**Total**	124(80.0%)	31(20.0%)	155(100.00%)			

## Discussion

Television was the second most common source of information regarding EVD in this study. However, in a similar study among nursing students in Nellore, only 10% of respondents obtained the information from television whereas majority of them, 53.3% obtained information from newspapers.^14^ In this study, only 36.2% of students obtained information about EVD from the internet compared to 80.4% of undergraduate students in a study in Malaysia who indicated the internet as the first source of information on the disease.^11^ In a related study in the United States of America, only 11% of respondents indicated “official government websites” as the source of information regarding EVD.^12^

Most of the respondents knew the correct mode of transmission of EVD, with as many as 71% correctly indicating one of the main modes of transmission. This is comparable to the findings of a similar study among students in Tehran, Iran where 74% of respondents knew the correct mode of transmission of the disease.^15^ About 12% of students in this study wrongly indicated that transmission of the disease can be prevented by taking chemoprophylaxis each time one touched a suspicious patient whereas in the Tehran study, as many as 46% of the respondents did not have any idea about how to prevent the transmission of the disease.^15^ In the study in the United States of America, 31% of respondents indicated that EVD can be transmitted by mosquitoes.^12^ As high as 41.2% of respondents did not indicate preparation and eating of infected bushmeat as a means of transmission of EVD. This may be a form of denial. Bushmeat is a highly valued delicacy in Cross River State. Bushmeat eating restaurants, referred to locally as “bush meat joints” where bushmeat is prepared with local scent leaves and served with palm wine and plantain, are common both in the rural and urban areas of the state. Some people in the state say they would rather die than forego their favourite delicacy (bushmeat), irrespective of whatever disease may be related to it.

Nearly 70% of respondents knew the correct incubation period of the disease. This proportion was comparable to the nearly 78% reported in a study among health care providers in Lagos state, Nigeria.^13^

Regarding the treatment of EVD, 71.8% of respondents in this study indicated that there is no known cure yet. This proportion was comparable to the 72.2% of respondents in Bayelsa state, Nigeria, who gave a similar response in a multi-site study in Nigeria.^16^ In the same multi-site study, only 41% of respondents from Kano gave the same response as the Bayelsa state respondents.^16^ Bayelsa and Cross River States are in Nigeria's Niger Delta region while Kano is in Northern Nigeria. A lot more social, economic and educational interactions, including sharing of knowledge and information, take place between residents of Bayelsa and Cross River States than with Kano state which is in the far North.

With respect to practice, this study found that only 45.8% of respondents indicated their unwillingness to work in the EVD treatment/isolation centre in the event of an EVD epidemic in Nigeria while 41.8% indicated their unwillingness to volunteer in EVD vaccine trial. These proportions are low when compared to the findings of the multi-site study in Kano, Bayelsa and Cross River States where 70% of health care workers in Calabar indicated their unwillingness to work in units caring for EVD patients.^16^ The difference may be explained by the fact that the respondents in our study are still in training to become health care providers (nurses) and may not be as afraid of contacting infectious diseases from patients as those who are already in practice which were the respondents in the multi-site study. Those who are already in practice may have seen practical cases of transmission of infections from patients to their health care providers while the students of nursing may only be hearing and reading about it theoretically.

Interestingly, only 7.9% of respondents indicated that studying in the same classroom with someone who has not manifested symptoms of EVD can lead to contacting the virus. Similarly, 7.9% of respondents indicated that if a roommate was suspected to have EVD, they would pack out of the room and return after they had been declared cured. These low proportions may be an indication that stigma and discrimination against people suspected of having EVD would be low. This also reflects in the finding that all the respondents (100%) indicated that if a friend who had EVD became certified cured, they would be willing to continue with the friendship. It also reflects in patient care as 76.3% indicated that if they knew that their patient who had come in labour had EVD but had been certified cured, they would deliver her of her baby. Similarly, 87.6% indicated their willingness to be part of the surgical operation of their patient who had been certified cured of EVD whereas in the study among healthcare providers in Lagos, Nigeria, only 43% of respondents indicated their willingness to join a volunteer corps of visiting physicians in providing health care to Ebola virus disease patients.^13^ Surprisingly, the good attitude of respondents in this study towards people cured of EVD did not reflect in their relationship with the school food vendor as only 52% of them indicated their willingness to patronize them if they were certified cured.

Nearly 16% of respondents indicated that one of the ways of preventing EVD is by addressing myths and misconceptions. This is very important because myths and misconceptions regarding health-related phenomena are very common in Nigeria. The belief in witches and wizards and evil spirits as the cause of virtually every health-related event influences the health-seeking behavior of the people. During the EVD outbreak in Nigeria, several myths and misconceptions circulated in the social media.^17^,^18^. This led to such unwholesome practices as having salt water bath and drinking salt water, eating of bitter kola, drinking one's urine, walking naked around the compound at midnight, consultation of spiritual medium, recitation of incantations and sacrificing to the gods, as the means of preventing and curing the disease. Myths and misconceptions regarding Ebola have also been reported by other researchers and reports.^19^,^20^,^21^ The WHO had indicated that bursting myths was crucial to stopping the transmission of EVD in Guinea.^22^

Only 32.2% of respondents in this study had attended workshop/training in EVD case management while 37.3% had attended workshop/training on EVD prevention. These proportions are high compared to the 16.6% of respondents in a study among health care providers in Lagos, Nigeria, who had received training on how to identify suspected EVD patients.^13^

## Conclusion

Majority of the students were knowledgeable about EVD. Although majority of them had favorable attitude towards EVD patients, there was evidence of stigmatizing and discriminatory attitude that need to be addressed. This is necessary in order to ensure that these students graduate to become providers of unbiased health care to all categories of patients.
